# Prediction of disease recurrence or residual disease after primary endoscopic resection of pT1 colorectal cancer—results from a large nationwide Danish study

**DOI:** 10.1007/s00384-023-04570-y

**Published:** 2023-11-30

**Authors:** Ilze Ose, Katarina Levic, Lau Caspar Thygesen, Orhan Bulut, Thue Bisgaard, Ismail Gögenur, Tine Plato Kuhlmann

**Affiliations:** 1 Center for Surgical Science, Department of Surgery, Zealand University Hospital, Lykkebækvej 1, 4600 Køge, Denmark; 2 Department of Surgical Gastroenterology, Copenhagen University Hospital, Hvidovre, Denmark; 3 National Institute of Public Health, University of Southern Denmark, Odense, Denmark; 4 Department of Surgical Gastroenterology, Holbæk Hospital, Holbæk, Denmark; 5 Department of Pathology, University Hospital of Copenhagen, Herlev Hospital, Herlev, Denmark

**Keywords:** pT1, Recurrence, Residual disease, Colorectal cancer

## Abstract

**Purpose:**

Risk assessment of disease recurrence in pT1 colorectal cancer is crucial in order to select the appropriate treatment strategy. The study aimed to develop a prediction model, based on histopathological data, for the probability of disease recurrence and residual disease in patients with pT1 colorectal cancer.

**Methods:**

The model dataset consisted of 558 patients with pT1 CRC who had undergone endoscopic resection only (*n* = 339) or endoscopic resection followed by subsequent bowel resection (*n* = 219). Tissue blocks and slides were retrieved from Pathology Departments from all regions in Denmark. All original slides were evaluated by one experienced gastrointestinal pathologist (TPK). New sections were cut and stained for haematoxylin and eosin (HE) and immunohistochemical markers. Missing values were multiple imputed. A logistic regression model with backward elimination was used to construct the prediction model.

**Results:**

The final prediction model for disease recurrence demonstrated good performance with AUC of 0.75 [95% CI 0.72–0.78], HL chi-squared test of 0.59 and scaled Brier score of 10%. The final prediction model for residual disease demonstrated medium performance with an AUC of 0.68 [0.63–0.72].

**Conclusion:**

We developed a prediction model for the probability of disease recurrence in pT1 CRC with good performance and calibration based on histopathological data. Together with lymphatic and venous invasion, an involved resection margin (0 mm) as opposed to a margin of ≤ 1 mm was an independent risk factor for both disease recurrence and residual disease.

## Introduction

Since the introduction of the Danish national colorectal cancer (CRC) screening program in 2014, there has been a threefold rise in the incidence of stage I CRC and according to data from the Danish Colorectal Cancer Group (DCCG.dk) 19.8% of newly diagnosed colon cancers in 2017 were pT1 cancers [1]. Unfortunately, it is still not perfectly clear how to manage early (pT1) CRC optimally. In many cases, major bowel resection with regional lymphadenectomy is performed, but this may be associated with a significant risk of post-operative mortality and morbidity, especially in elderly or fragile patients. Endoscopic local excision of pT1 CRC is a less invasive, organ-preserving procedure that may be an especially attractive option for patients with significant comorbidity and frailty. The risk of disease recurrence after resection of pT1 cancer ranges between 2 and 10% [2–4], depending heavily on several histopathological risk factors. Seven to 20% of all pT1 CRC patients will have lymph node metastasis (LNM) at the time of diagnosis [5, 6] and the oncological outcomes are only comparable to major bowel resection with regional lymphadenectomy when LNM are absent [7]. Moreover, approximately 1.8–3% of patients with T1 cancer will develop distant metastasis [2, 8]. Today, when local excision is performed for pT1 CRC, pathologists play a crucial role in stratifying patient risk by examining histopathological risk factors. Over the years, several risk factors for lymph node metastasis have been reported [9, 10]. Many of these risk factors correlate not only with the risk of LNM but also with risk of distant metastasis and thereby to the overall risk of disease recurrence [3, 8]. However, this single parameter-based risk assessment overestimates the risk of LNM, as 80–90% of the patients selected for additional surgery will have no evidence of LNM or residual disease [11, 12]. Several risk scores and prediction models have already been proposed [3, 13–15]. Nevertheless, clinical multicentre studies are still needed to fully confirm the predictive value of histopathological risk factors, not only their role in predicting LNM, but also their impact on overall disease recurrence, despite the pathophysiological variations in recurrence mechanisms. The current study aimed to develop a prediction model based on histopathological data for the probability of disease recurrence and residual tumour in a large, nationwide cohort of Danish patients with pT1 CRC.

## Materials and methods

### Study design

This is a nationwide retrospective cohort study of patients diagnosed with pT1 CRC between January 2001 and December 2011. The Data Protection Agency in Denmark and the Medical Ethics Committee of the Capitol Region in Denmark approved the study (Approval ID: 2013–41–2475 and H-15001716). This study was performed in accordance with the Helsinki Declaration.

### Study population

Patients over the age of 17 who underwent endoscopic resection (ER) of pT1 CRC with or without subsequent bowel resection (SBR) between January 2001 and December 2011 were retrospectively evaluated. Only patients without previous surgery for colorectal cancer who underwent complete endoscopic resection of pT1 CRC were included in the study. Patients diagnosed with Lynch syndrome, familial adenomatous polyposis, patients with active inflammatory bowel disease, multiple malignant lesions or synchronous tumours were excluded. Patients were also excluded if histological blocks or endoscopy reports were missing, if the histological re-evaluation revealed non-invasive lesions or if the patients had received neoadjuvant radiotherapy. ER included endoscopic mucosal resection (EMR), endoscopic submucosal dissection (ESD) and snare polypectomy. pT1 CRC was defined as adenocarcinomas invading through the muscularis mucosae into the submucosa, but not involving the muscularis propria [16]. During the study period between January 2001 and December 2011, guidelines from the Danish Colorectal Cancer Group (DCCG.dk) recommended subsequent surgery if at least one of the following risk factors were present: positive resection margin (< 1 mm), poorly differentiated adenocarcinoma or lymphovascular invasion [17]. SBR was performed as an open or laparoscopic procedure.

### Data source

The patients were identified from the Danish Colorectal Cancer Group (DCCG.dk) database. The data gathered were supplemented with data from the Danish National Patient Register (NPR) and the Danish National Pathology Register and Data Bank (DNPR) [18–20]. All data were crosschecked with manual reviews of medical, endoscopy, pathology reports and radiology charts and additional information on patient and tumour characteristics were collected. All available paraffin blocks and haematoxylin and eosin (HE)-stained sections on primary confirmed cases of pT1 CRC were retrieved from nationwide Pathology Departments. Patients were followed up until December 2016 or until death.

### Pathological evaluation

HE staining was used as standard for the histopathological re-evaluation. In all available cases, the original HE slides were re-evaluated to confirm or reject the diagnosis of pT1 CRC. In case of missing original HE slides, new sections were cut from all available blocks and stained for HE. From each case, one or two blocks were selected for inclusion in the study. On the included material, both control HE and immunohistochemical staining was performed: cytokeratin (CKAE1/AE3), D2-40, caldesmon, pMLH1, pMSH2, pMSH6 and pPMS2. All original HE slides, new HE- and immunohistochemical stained slides from the included cases were re-evaluated by an experienced pathologist subspecialised in gastrointestinal pathology (TPK). She was blinded for the results from the original pathology reports and clinical characteristics, except for the endoscopic polyp type (pedunculated or sessile) and whether the polyp had been completely removed in one piece or was removed by piece-meal technique. The following data were recorded at the re-evaluation of each case: tumour type defined according to WHO 2019 [16]. Presence of mucinous tumour component. Invasive tumour size: measured as the largest diameter at the invasive front in mm. Tumour level: Haggitt level 1–4 for pedunculated polyps, Kikuchi level Sm1–3 for sessile polyps [21, 22]. Tumour grade: low grade and high grade, based on the worst area of differentiation. Distance from invasive tumour to the resection margin, measured in mm: 0 mm (involved margin), ≤ 1 mm, > 1 mm. Perineural invasion, intramural lymphatic invasion (HE and D2-40 staining) and intramural venous invasion (HE and caldesmon staining). Tumour budding: Bd1 (0–4 buds), Bd2 (5–9 buds) or Bd3 (≥ 10 buds). Tumour budding was defined as “a single cancer cell or a cell cluster of up to four tumour cells” and counted according to the recommendations of the International Tumour Budding Consensus Conference 2016 (scored on HE, if necessary guided by CK staining) [23]. Mismatch repair protein (MMR) status: pMLH1, pMSH2, pMSH6 and pPMS2.

### Outcomes

The primary outcome was to develop a prediction model for disease recurrence in patients with pT1 CRC. Patients who underwent complete ER without SBR and developed locoregional and/or distant CRC recurrence during a 5-year follow-up period and patients who underwent complete ER followed by SBR with ≥ 1 positive lymph node in the resection specimen or developed distant CRC recurrence during a 5-year follow-up period were defined as disease recurrence-positive cases. Locoregional recurrence was defined as any recurrent tumour growth or recurrences in lymph nodes near the primary resection site. Distant recurrence was defined as any histological, morphological and clinical evidence of metastasis in distant organs, bones or peritoneum.

The secondary outcome was to develop a prediction model for residual disease in patients with pT1 CRC after primary endoscopic resection. Residual disease was defined as histologically verified tumour tissue in the mucosa and bowel wall at the primary resection site following SBR.

### Candidate variables for predicting disease recurrence

Based on previous literature and current guidelines, a set of candidate variables for predicting disease recurrence were selected. These included tumour grade, polyp shape, polyp size, distance to the resection margin, high risk (Haggitt level 3–4 or Kikuchi Sm3), intramural venous invasion, lymphatic invasion and the tumour budding score (Bd1–3).

### Statistical analysis

Categorical variables were summarised as counts and percentages; medians (interquartile ranges; IQR) were used for continuous variables. Multiple imputations by fully conditional specification (FCS) method were used for missing data by imputing 20 data sets using the SAS procedure PROC MI [24]. Univariate and multivariate analysis of disease recurrence and residual disease was performed by logistic regression model and reported as odds ratio (OR) with a 95% confidence interval (CI). Backward selection using a liberal significance level of 0.157 was used to select the prediction model. Since we used multiple imputations, the selection method was conducted in all data sets, and we included variables selected in at least 10 analyses. The model performance was assessed for calibration and discrimination capability. Calibration was assessed using the Hosmer–Lemeshow (HL) goodness-of-fit test and the scaled Brier score. ROC curves and the corresponding area under the ROC curve (AUC) were calculated to test for discrimination [25]. Statistical analyses were conducted using SAS version 9.4. All reporting was conducted in accordance with the STROBE statement.

## Results

### Study population

**Fig. 1:**
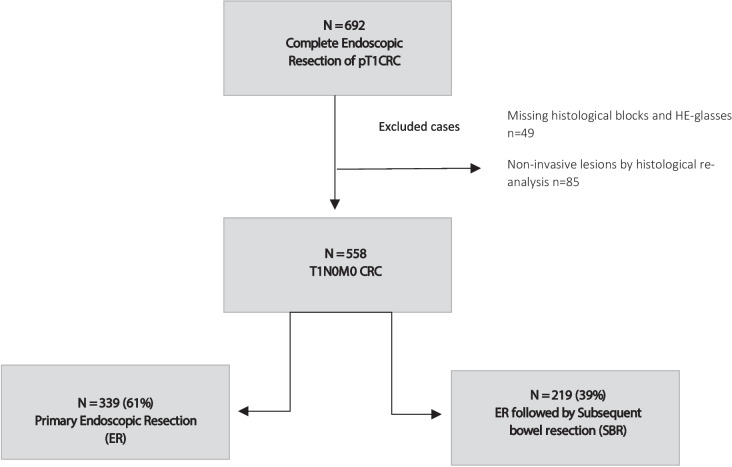
Flow chart illustrating the study population. CRC colorectal cancer

A total of 692 patients with pT1 CRC were identified through the DCCG.dk database. Paraffin blocks and HE slides from 49 patients could not be retrieved, and they were excluded from the analysis. After the histopathological re-evaluation of the original HE slides, another 85 patients were excluded from further analysis, due to either rejection of the primary diagnosis of adenocarcinoma or if the diagnosis was uncertain based on the available material. The final cohort consisted of 558 patients. Among these, 339 patients (61%) underwent complete endoscopic resection (ER), and 219 patients (39%) underwent ER and subsequent bowel resection (ER + SBR). Figure 1 shows the study flow chart. The median follow-up time of the study group ER and ER + SBR were 79.0 months (IQR 55.5–112.0 months) and 96.0 moths (IQR 71.0–122.5 months), respectively. Baseline clinical and histopathological characteristics are shown in Table 1.

**Table 1:** Baseline clinical and histopathological characteristics

	**Endoscopic resection (ER)** ***N* = 339**	**ER + subsequent bowel resection** ***N* = 219**
**Age, years, median (IQR)**	73 ± 16	65 ± 15.5
**Sex, *n* (%)**		
Male	200 (59.0)	116 (53.0)
Female	139 (41.0)	103 (47.0)
**BMI (kg/m**^**2**^**), median (IQR)**	25 ± 5.3	26 ± 5.2
**Charlson comorbidity index (CCI)**		
0	224 (66.1)	165 (75.3)
1–2	93 (27.4)	39 (17.8)
≥3	22 (6.5)	15 (6.8)
**Location, *n* (%)**		
Colon	228 (67.2)	167 (76.3)
Rectum	111 (32.7)	52 (23.7)
**Polyp shape, *n* (%)**		
Pedunculated	238 (70.2)	127 (58.0)
Sessile	67 (19.8)	74 (33.8)
Missing	34 (10.0)	18 (8.2)
**Polyp size, mm, median (IQR)**	17 ± 13.0	18 ± 11.0
**Polypectomy technique, *n* (%)**		
En bloc resection	273 (80.5)	159 (72.6)
Piecemeal resection	66 (19.5)	60 (27.4)
**Tumour grade, *n* (%)**		
Low grade	304 (89.7)	181 (82.6)
High grade	25 (7.4)	23 (10.5)
Cannot be evaluated	10 (2.9)	15 (6.8)
**Type of carcinoma, *n* (%)**		
Adenocarcinoma	330 (97.3)	201 (91.8)
Mucinous adenocarcinoma	9 (2.7)	18 (8.2)
**Resection margin, *n* (%)**		
Negative (> 1 mm)	156 (46.0)	55 (25.1)
Positive (≤ 1 mm)	130 (38.3)	121 (55.3)
Cannot be evaluated	53 (15.6)	44 (20.1)
**Resection margin, *n* (%)**		
0 mm	44 (13.0)	61 (27.9)
>0 mm	242 (71.4)	114 (52.1)
Cannot be evaluated	53 (15.6)	44 (20.1)
**Mucinous component, *n* (%)**		
Absent	317 (93.5)	201 (91.8)
Present	19 (5.6)	12 (5.5)
Missing	<5	6 (2.7)
**Haggitt classification, *n* (%)**		
Level 1–2	74 (21.8)	23 (10.5)
Level 3–4	127 (37.5)	66 (30.1)
Cannot be evaluated	73 (21.5)	58 (26.5)
**Kikuchi classification, *n* (%)**		
Sm1–Sm2	25 (7.4)	9 (4.1)
Sm3	26 (7.7)	44 (20.1)
Cannot be evaluated	14 (4.1)	16 (7.3)
**High risk (Haggitt level 3–4 or Kikuchi Sm3)**		
No	100 (29.5)	33 (15.1)
Yes	154 (45.4)	110 (50.2)
Cannot be evaluated	85 (25.1)	76 (34.7)
**Intramural venous invasion, *n* (%)**		
Absent	266 (78.5)	149 (68.0)
Present	56 (16.5)	43 (19.6)
Cannot be evaluated	17 (5.0)	27 (12.4)
**Lymphatic invasion, *n* (%)**		
Absent	240 (70.8)	123 (56.2)
Present	84 (24.8)	75 (34.2)
Cannot be evaluated	15 (4.4)	21 (9.6)
**Perineural invasion, *n* (%)**		
Absent	318 (93.8)	194 (88.6)
Present	<6	<6
Cannot be evaluated	16 (4.7)	24 (11.0)
**Tumour budding, *n* (%)**		
Yes	232 (68.4)	138 (63.0)
No	40 (11.8)	13 (6.0)
Cannot be evaluated	67 (19.8)	68 (31.0)
**Budding level, *n* (%)**		
Bd1	179 (52.8)	89 (40.6)
Bd2–3	53 (15.6)	41 (18.7)
Cannot be evaluated	108 (31.9)	89 (40.6)
**pMLH1, *n* (%)**		
No expression	<6	<6
Normal expression	326 (96.2)	207 (94.5)
Heterogeneous expression	8 (2.3)	<6
Cannot be evaluated	<6	<6
**pMSH2, *n* (%)**		
No expression	<6	<6
Normal expression	326 (96.2)	209 (95.4)
Heterogeneous expression	8 (2.3)	<6
Cannot be evaluated	<6	<6
**pMSH6, *n* (%)**		
No expression	<6	<6
Normal expression	327 (96.5)	209 (95.4)
Heterogeneous expression	8 (2.3)	<6
Cannot be evaluated	<6	<6
**pPMS2, *n* (%)**		
No expression	<6	<6
Normal expression	326 (96.2)	207 (94.5)
Heterogeneous expression	8 (2.3)	<6
Cannot be evaluate	<6	<6
**Endoscopic complication, *n* (%)**		
Yes	11 (3.2)	<6
Bleeding	10	<6
Reoperation	<6	<6
No	328 (96.8)	213 (97.3)
**Surgical approach, *n* (%)**		
Open	–	95 (43.4)
Laparoscopic	–	124 (56.6)
Conversion to open surgery		18 (14.5)
**Surgical complication, *n* (%)**		
Intraoperative complications	–	8 (3.7)
Postoperative surgical complications	–	
Yes	–	41 (18.7)
No	–	178 (81.3)
Postoperative medical complications	–	
Yes	–	16 (7.3)
No	–	203 (92.7)
**Postoperative complications according to CD classification, *n* (%)**		
CD grade I		<6
CD grade II		10 (17.59)
CD grade IIIa		<6
CD grade IIIb		19 (33.3)
CD grade IV		8 (14.0)
CD grade V		9 (15.8)

*BMI* body mass index, *ASA* American Society of Anaesthesiology, *CD* Clavien–Dindo classification

### Disease recurrence and residual disease

A total of 27 patients (8.0%) in the ER group experienced disease recurrence. Among them, 12 patients (3.5%) were diagnosed with locoregional recurrence, and 15 patients (4.5%) developed distant metastasis. In contrast, a significantly higher number of patients, 34 (15.5%), in the ER + SBR group developed disease recurrence, *p* = 0.008. A total of 15 (11.9%) had positive lymph nodes in the resection specimen after SBR. The pathology reports of the resection specimens revealed 11 (5.0%) cases in which the pathological T-category was higher than pT1. These cases were excluded from further analysis. A total of 8 (3.7%) patients in the ER + SBR group developed distant metastasis during the follow-up period. There was no significant difference in the proportion of distant metastases between the ER group and the ER + SBR group, *p* = 0.68. Finally, 50 (8.1%) disease recurrence positive cases were used for the development of the clinical prediction model for disease recurrence. The presence of residual disease was identified in 21 (9.6%) cases after SBR. Table 2 shows the rates of disease recurrence and residual disease in the study population.

**Table 2:** The rate of disease recurrence and residual disease

	**ER**	**ER+SBR**	***p* value**
**Disease recurrence, *n* (%)**	27 (8.0)	34 (15.5)	0.008
LNM positive after SBR	–	15 (11.9)	
Locoregional recurrence	12 (3.5)	–	
Distant metastases	15 (4.4)	8 (3.7)	0.68
Upstaged (> T1) CRC after SBR	–	11 (5.0)*	
**Residual disease, *n* (%)**			–
Yes	–	21 (9.6)	
No	–	198 (90.4)	

*LNM* lymph node metastasis

*Excluded from further analysis

### Derivation of the prediction model for disease recurrence

As described previously, 50 (8.1%) cases were identified positive for disease recurrence. The logistic regression analysis is illustrated in Table 3.

**Table 3:** Univariate and multivariate logistic regression analysis for disease recurrence

**Clinicopathological factors**	**Univariate analysis** **OR (95% CI), *p* value**	**Multivariate analysis** **OR (95% CI), *p* value**
**Tumour grade**		
Low grade	1.00 (ref)	1.00 (ref)
High grade	2.69 (1.22–5.97), 0.01	1.32 (0.48–3.62), 0.59
**Polyp shape**		
Sessile	1.00 (ref)	1.00 (ref)
Pedunculated	1.02 (0.51–2.04), 0.96	1.43 (0.64–3.23), 0.39
**Polyp size, mm**		
<4	1.00 (ref)	1.00 (ref)
≥4	1.76 (0.57–5.47), 0.33	0.79 (0.22–2.87), 0.72
**Resection margin, mm**		
>0	1.00 (ref)	1.00 (ref)
0	3.44 (1.77–6.68), 0.0003	2.45 (1.10–5.48), 0.03
**Resection margin, mm**		
>1	1.00 (ref)	1.00 (ref)
0–1	1.67 (0.85–3.28), 0.14	0.98 (0.44–2.22), 0.97
**High risk (Haggitt level 3–4 or Kikuchi Sm3)**		
No	1.00 (ref)	1.00 (ref)
Yes	6.02 (1.46–24.9), 0.01	3.34 (0.69–16.2), 0.14
**Intramural venous invasion**		
No	1.00 (ref)	1.00 (ref)
Yes	3.35 (1.67–6.70), 0.008	3.04 (1.41–6.53), 0.005
**Lymphatic invasion**		
No	1.00 (ref)	1.00 (ref)
Yes	3.67 (1.88–7.14), 0.0007	2.54 (1.18–5.47), 0.02
**Budding level**		
Bd1	1.00 (ref)	1.00 (ref)
Bd2–3	2.99 (1.35–6.62), 0.08	1.44 (0.55–3.75), 0.46

After backward model selection, the following variables remained in the final model: resection margin with a cut-off point of 0 mm [OR, 2.84; 95% CI, 1.39 to − 5.79; *p* = 0.004], presence of intramural venous invasion [3.12; 1.52–6.42; *p* = 0.002] and lymphatic invasion [3.34; 1.67–6.68; *p* = 0.002]. Table 4 illustrates variables selected for the prediction model after backward selection.

**Table 4:** Variables selected after backward selection for the prediction model for disease recurrence

**Clinicopathological factors**	**Multivariate analysis** **OR (95% CI), *p* value**	**OR min max** **Imputed data set (*n* = 20 × 558)**
**Resection margin, mm**		
>0	1.00 (ref)	
0	2.84 (1.39–5.79), 0.004	2.19–4.10
**Lymphatic invasion**		
No	1.00 (ref)	2.77–4.98
Yes	3.34 (1.67–6.68), 0.0007	
**Intramural venous invasion**		
No	1.00 (ref)	
Yes	3.12 (1.52–6.42), 0.002	2.21–4.27

**Fig. 2:**
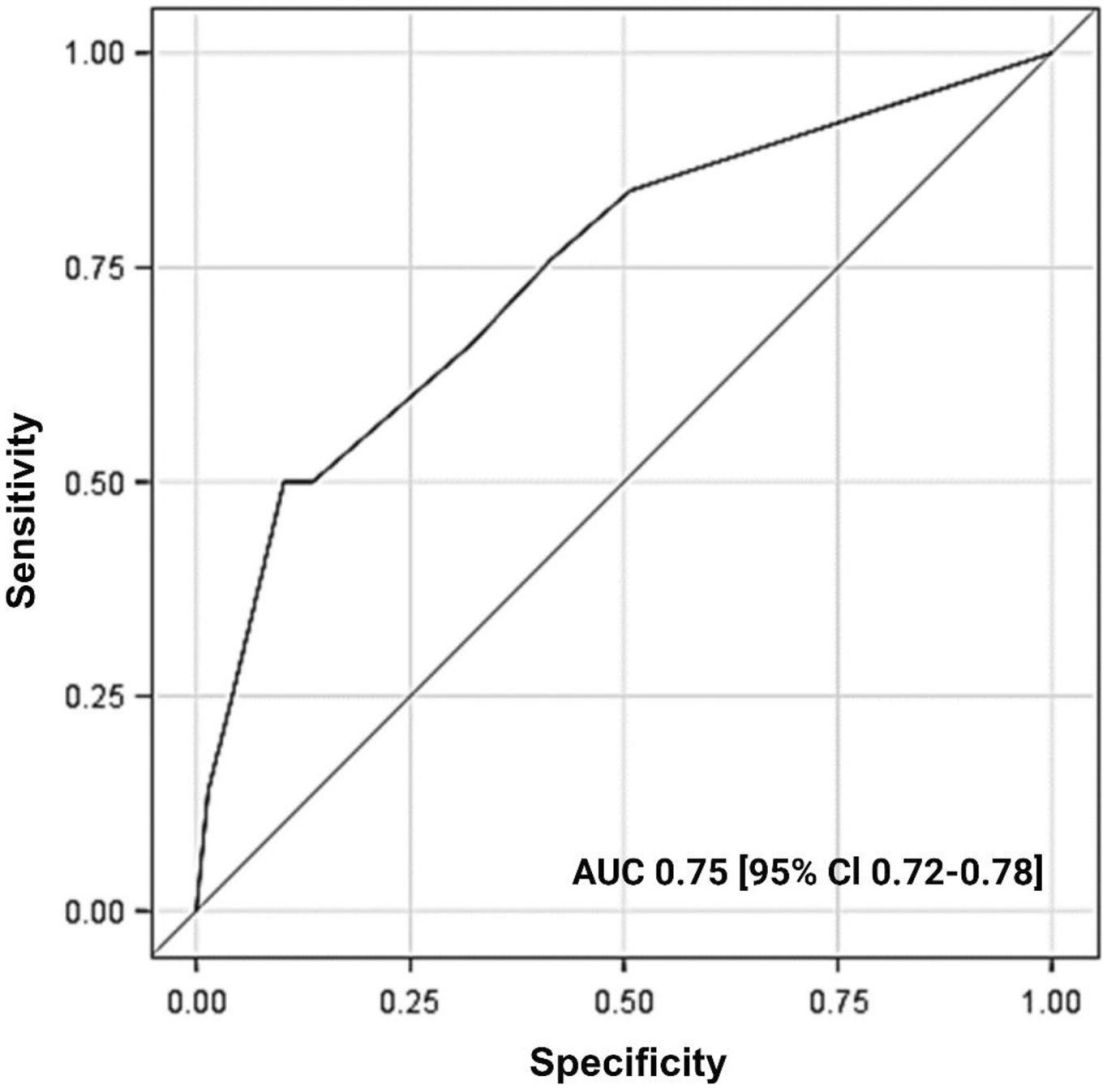
Receiver operating characteristic curve (AUC) of the predictions model for disease recurrence

The model demonstrated good performance for the prediction of disease recurrence (AUC = 0.75; 95% CI, 0.72–0.78; scaled Brier score = 10%). Figure 2 shows the ROC curve for disease recurrence prediction. The Hosmer–Lemeshow goodness-of-fit test yielded a *p* value of 0.59, suggesting good agreement between observed and predicted numbers of disease recurrence.

### Derivation of the prediction model for residual disease

A total of 21 patients had residual disease after SBR. The prediction model was constructed using the same methodology as the disease recurrence prediction model. Univariate and multivariate logistic regression analysis is illustrated in Table 5.

**Table 5:** Univariate and multivariate logistic regression analysis for residual disease

**Clinicopathological factors**	**Univariate analysis** **OR (95% CI), *p* value**	**Multivariate analysis** **OR (95% CI), *p* value**
**Tumour grade**		
Low grade	1.00 (ref)	1.00 (ref)
High grade	0.45 (0.06–3.53), 0.45	0.27 (0.03–5.59), 0.26
**Polyp shape**		
Sessile	1.00 (ref)	1.00 (ref)
Pedunculated	0.97 (0.36–2.61), 0.95	1.21 (0.41–3.53), 0.73
**Polyp size, mm**		
<4	1.00 (ref)	1.00 (ref)
≥4	NA	NA
**Resection margin, mm**		
>0	1.00 (ref)	1.00 (ref)
0	3.16 (1.19–8.36), 0.02	3.01 (1.07–8.46), 0.04
**Resection margin, mm**		
>1	1.00 (ref)	1.00 (ref)
0–1	3.03 (0.49–18.9), 0.24	2.86 (0.40–20.3), 0.30
**High risk (Haggitt level 3–4 or Kikuchi Sm3)**		
No	1.00 (ref)	1.00 (ref)
Yes	NA	NA
**Intramural venous invasion**		
No	1.00 (ref)	1.00 (ref)
Yes	1.66 (0.57–4.78), 0.35	1.37 (0.43–4.35), 0.60
**Lymphatic invasion**		
No	1.00 (ref)	1.00 (ref)
Yes	1.31 (1.46–3.70), 0.61	1.18 (0.33–4.19), 0.80
**Budding level**		
Bd1	1.00 (ref)	1.00 (ref)
Bd2–3	2.25 (0.83–6.05), 0.11	2.20 (0.65–7.47), 0.21

After backward model selection, only resection margin with a cut-off point of 0 mm [OR, 2.91; 95% CI, 1.07–7.94; *p* = 0.04] was included in the model. Budding level Bd2–3 was nearly significant [1.96; 0.70–5.52; *p* = 0.20] and was present in 8 of 20 imputed data sets. Due to the absence of other relevant variables, we included the budding level in the final prediction model. Table 6 illustrates variables selected for the prediction model after backward selection.

**Table 6:** Variables selected after backward selection for the prediction model for residual disease

**Clinicopathological factors**	**Multivariate analysis** **OR (95% CI), *p* value**	**OR min max** **Imputed data set (*n* = 20 × 558)**
**Resection margin, mm**		
>0	1.00 (ref)	
0	2.91 (1.07–7.94), 0.04	2.02–3.99
**Budding level**		
Bd1	1.00 (ref)	
Bd2–3	1.96 (0.70–5.52), 0.20	1.46–307

**Fig. 3:**
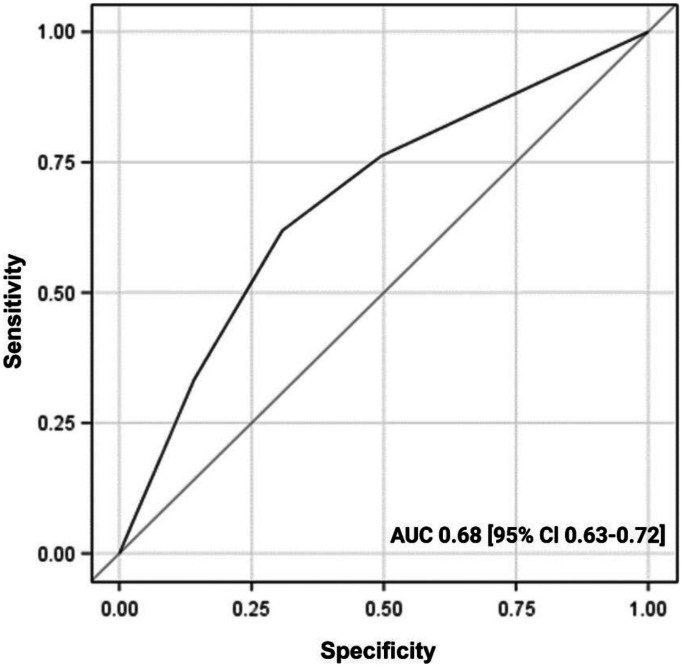
Receiver operating characteristic curve (AUC) of the predictions model for residual disease

The ROC curve demonstrated medium performance of the prediction model with an AUC of 0.68 (95% CI, 0.63–0.72). Figure 3 shows the ROC curve for residual disease prediction. The Hosmer–Lemeshow goodness-of-fit test had a *p* value of 0.77 and a scaled Brier score of 3%.

## Discussion

The aim of the present study was to develop a prediction model for disease recurrence and residual disease based on histopathological factors in patients with pT1 CRC. We identified 50 (8.1%) disease recurrence positive cases in our data set. Intramural venous invasion, lymphatic invasion and a positive resection margin (involved margin) were all independent predictive factors for disease recurrence. Consequently, these variables were selected for the prediction model for disease recurrence. The model performance was good in terms of discrimination and calibration. Furthermore, we developed a prediction model for residual disease. Multivariate analysis identified a positive (involved) resection margin as an independent predictive factor, and additionally, we included tumour budding Bd2–3 in the prediction model, despite borderline significance. The model demonstrated medium performance for discriminating patients with residual disease, most likely due to the small sample size of the dataset available for model derivation.

Among patients who underwent subsequent bowel resection, more than 80% had no LNM in the subsequent surgical specimen, which perfectly demonstrates the challenges in distinguishing between high- and low-risk pT1 CRC patients. The prevalence of LNM and distant metastases in the current study was in accordance with the existing literature [26]. Similar to our study, lymphovascular invasion is one of the most reliable predictors for LNM in pT1 CRC in many studies [27]. However, previous studies have underlined that these should be recorded separately, as done in our study, since the presence of submucosal lymphatic invasion and to a lesser degree venous invasion are some of the strongest predictors of LNM in pT1 CRC [28]. In contrast to the above, we found similar odds ratios for both lymphatic invasion and vascular invasion. The recognition of both lymphatic and vascular invasion can be difficult, as lymphatics can be hard to distinguish from venules, and other factors like retraction artefacts, tumour budding or poorly differentiated clusters may further complicate the picture. Consequently, the histopathological evaluation of lymphatic invasion is known to be subjective with significant rates of inter-observer variation [29]. Compared to several other studies, the presence or absence of both lymphatic and venous invasion in the current study was confirmed by immunohistochemistry for D2-40 and caldesmon, respectively. The use of immunohistochemistry has been shown to increase both the number of detected cases and to significantly improve the inter-observer agreement [30].

The Kikuchi and Haggitt classification is used for risk stratification of lymph node metastasis in several international guidelines, including the current Danish guidelines [31]. In accordance with the challenges described in the literature, regarding the use of Kikuchi and Haggitt classification, the level of invasion could not be evaluated in 11.4% and 48% of cases, respectively, during histopathological re-evaluation. This limitation hinders the accurate determination of the extent of tumour invasion and, consequently, the ability to make informed decisions regarding subsequent treatment [32]. As of today, there is still significant controversy about the degree of risk of local recurrence, lymph node metastasis and distant metastasis in cases where a tumour extends close to the deep resection margin (1 mm or less) but does not directly involve it. In the current Danish guidelines, a resection margin distance of > 1 mm is still recommended [33], but also in Denmark the discussion of the cut-off for positive margin is ongoing. Some studies have reported that a resection margin > 0 mm, in the absence of other histological risk factors, effectively identifies patients at low risk of residual disease and lymph node metastases [34, 35]. In the current study, we included both resection margins with a 0 mm cut-off value (involved margin) and a 1 mm cut-off value as a predictor for disease recurrence and residual disease. Interestingly, only resection margin with a cut-off point of 0 mm qualified for inclusion in the final prediction model.

Previous studies have reported prediction models for both, LNM and distant metastasis, based on histopathological factors with results similar to our study [8, 15, 36]. Recently, prediction models developed by artificial intelligence (AI) methods and AI-aided histopathological evaluation have demonstrated stronger performance than that of conventional models [37, 38]. Aside from the fact that these models are not yet fully integrated into clinical practice, one of their limitations is that some rely solely on histopathology reports rather than digital histopathology slides. Furthermore, the current AI models for detecting LNM are based on a sensitivity level of 100%, which may also present certain limitations. As a result, only a few extra unnecessary bowel resections could be potentially avoided compared to the use of histopathological risk factors as we know them today.

Overall, a common limitation of most studies on prediction of disease recurrence in pT1 CRC is restricted information on histopathological factors, heterogeneity in surgical procedures, small sample size and single-centre data. Our study has significant strengths compared to some of these earlier published studies, including the use of nationwide, validated patient data, including patients who underwent both only ER and ER with SBR with sufficiently long follow-up time, and the fact that the predictive model is based on re-evaluation of all cases by one experienced pathologist and not only on pre-existing pathology reports.

However, the study also has several limitations. The limited sample size and a low number of patients with disease recurrence and residual disease may introduce bias. Handling missing data poses inherent challenges, and the use of imputation introduces the potential for different final models in each imputed dataset. To mitigate this challenge, a suggested solution involves including variables that consistently appear in the final model. However, it is crucial to acknowledge that this method does not guarantee the relevance or stability of variables. A notable limitation of backward elimination is that once a variable is rejected, it is not re-entered. However, a rejected variable may become significant in the final model. We did not perform internal validation by data splitting into training and testing models, since independent validation would be misleading due to absence of sufficient sample size [39, 40]. Finally, we cannot determine the generalisability of the prediction model since our prediction model has not been externally validated.

In conclusion, while our prediction model for residual disease failed to demonstrate good performance, we succeeded in developing a prediction model for disease recurrence with good performance and calibration based on histopathological data. A unique result of this study is the finding of an involved resection margin (0 mm) as opposed to a margin of ≤ 1 mm, as an independent risk factor for both disease recurrence and residual disease. This finding might impact the coming Danish recommendations for the optimal treatment of patients with pT1 CRC.

## Data Availability

The data that support the findings of this study are available in an anonymous form from the corresponding author, IO, upon reasonable request.
